# Interleukin‐2 Deprived State of Regulatory T cells and Their Recovery by Low‐Dose Interleukin‐2 in Patients With Inflammatory Myopathies

**DOI:** 10.1002/art.70151

**Published:** 2026-04-28

**Authors:** Justus Ohmes, Luisa R. Monne, Sara Comdühr, Fynn Gerlach, Hanna Grasshoff, Alexander Dübbers, Antje Müller, Alexander Scheffold, Gabriela Riemekasten, Reza Akbarzadeh, Jens Y. Humrich

**Affiliations:** ^1^ Department of Rheumatology and Clinical Immunology University Medical Center Schleswig‐Holstein / University of Lübeck Lübeck Germany; ^2^ Institute of Immunology University Medical Center Schleswig‐Holstein / Christian‐Albrechts‐University Kiel Germany

## Abstract

**Objective:**

Regeneration and expansion of Treg by low‐dose interleukin‐2 (IL‐2) therapy is considered a potential treatment strategy for a wide range of autoimmune diseases. To provide a pathophysiologically‐based rationale for low‐dose IL‐2 therapy, we investigated whether reversible defects in the Treg–IL‐2 axis emerge in inflammatory myopathies.

**Methods:**

CD4+ T cell subsets from patients with polymyositis (PM) or dermatomyositis (DM) (n = 20) and healthy controls (HC) (n = 19) and peripheral blood mononuclear cells (PBMC) from eight patients that were stimulated in vitro for 24 hours with different concentrations of recombinant human IL‐2 were analyzed by multicolor flow cytometry. Messenger RNA (mRNA) expressions of *IL2RA*, *IL2RB*, *IL2RB*, *ENTPD1*, *IKZF2*, and *CTLA4* were quantified by real‐time polymerase chain reaction in IL‐2 stimulated PBMC (n = 6). Two patients with refractory PM/DM were treated with low‐dose IL‐2 therapy for eight weeks and monitored for clinical responses and changes in Treg subsets.

**Results:**

Frequencies of Treg expressing CD25 at high levels (CD25hi Treg) and of CXCR5+ Treg were reduced in patients with PM/DM compared with HC (*P* = 0.0052; *P* = 0.0661), particularly in active myositis (*P* = 0.0036; *P* = 0.0335). Stimulation with low doses of IL‐2 selectively enhanced expression of CD25 molecules by Treg, leading to an increase in the CD25hi Treg subset (*P* < 0.05) and augmented mRNA expression of several immunoregulatory molecules (*P* < 0.05). Low‐dose IL‐2 therapy induced decreases in muscle enzymes that were accompanied by a sustained expansion of CD25+ Treg.

**Conclusion:**

Our data suggest that shortage of IL‐2 is pathophysiologically relevant in PM/DM. Recovery and expansion of Treg by low‐dose IL‐2 therapy could thus be a promising targeted treatment option.

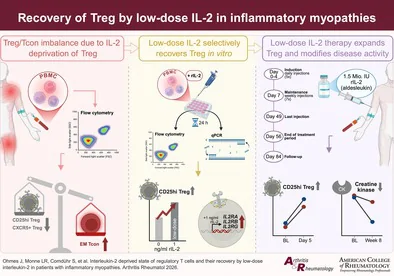

## INTRODUCTION

Polymyositis (PM) and dermatomyositis (DM) constitute the main entities within inflammatory myopathies (IM), a heterogeneous group of systemic autoimmune and connective tissue diseases that are characterized by chronic inflammation of the muscles and multiple other organs, such as the skin, lungs, and heart, causing organ dysfunction and increased morbidity, disability, and death.^1^, ^2^ Because the pathomechanisms are complex and not yet fully understood, the treatment of IM remains challenging, and apart from intravenous immunoglobulins (IVIG), no approved therapies exist.^3^ In systemic autoimmune diseases, including IM, a failure of peripheral immune tolerance is the fundamental cause of immune dysregulation and subsequent chronic inflammation. In PM/DM, the breach of tolerance against diverse nuclear and cytoplasmic autoantigens results in aberrant activation of autoreactive T and B cells, leading to the production of autoantibodies and tissue inflammation.^1^, ^3^ CD4+FoxP3+ Treg are crucial components of peripheral immune tolerance and essential for controlling autoimmunity by suppressing the activation and expansion of autoreactive and other harmful cells.^4^, ^5^, ^6^ Continued interleukin (IL)‐2 signaling is essential for Treg survival, growth, suppressive function, and lineage stability. Consequently, dysregulation in IL‐2 signaling or shortage of IL‐2 impairs Treg homeostasis and facilitates uncontrolled hyperactivity of autoreactive T and B cells, thereby promoting autoimmunity.^7^, ^8^, ^9^, ^10^, ^11^, ^12^, ^13^

Our previous studies provided evidence for a reversible impairment in Treg homeostasis in systemic lupus erythematosus (SLE) in both mice and humans that is related to an acquired decline in IL‐2 production. These findings built the pathophysiologic basis for a targeted intervention aiming to recover and expand Treg directly in patients by using low‐dose IL‐2 therapy.^11^, ^12^, ^14^, ^15^, ^16^, ^17^, ^18^, ^19^, ^20^ Typical hallmarks of an IL‐2 deprived state of Treg are the loss of the highly suppressive CD25hi Treg subsets and an imbalance between Treg and conventional T cells (Tcon).^14^, ^15^, ^16^, ^21^ To date, it remains unclear whether a relevant defect in the Treg‐IL‐2 axis is also involved in other connective tissue diseases apart from SLE. Here, we investigated whether Treg from patients with PM/DM display typical features of an IL‐2 deprived state and whether the CD25hi Treg population can be recovered by in vitro stimulation with low doses of IL‐2. To provide a first translational proof‐of‐concept, we investigated whether low‐dose IL‐2 therapy is capable of efficiently and safely restoring Treg homeostasis and influencing the disease course in two patients with refractory PM/DM.

## MATERIAL AND METHODS

### Study participants

For immunophenotyping, 12 patients with PM and 8 patients with DM (n = 20 in total) were consecutively recruited between 2019 and 2021 from the in‐ and outpatient care unit of the Department of Rheumatology and Clinical Immunology at the University Medical Center Schleswig‐Holstein in Lübeck, Germany. PM/DM was diagnosed in accordance with the American College of Rheumatology (ACR)/EULAR classification criteria from 2017.^22^ Blood samples were obtained from patients and from age‐ and sex‐matched healthy controls (HC) (n = 19) after providing informed consent. Clinical characteristics were retrieved from the patient's medical records (summarized in Supplementary Tables 1 and 2), and routine laboratory analyses and immunophenotyping were performed at the time of blood collection. Patients with active myositis were defined by having elevated plasma levels of the creatine kinase (CK) above the upper limit of normal of 170 U/L for women and 190 U/L for men (patients assessable for activity: n = 19). HC and patients having an ongoing infection or evidence of relevant drug‐induced toxicities were not included in the study. These studies were approved by the institutional review board at the University of Lübeck (AZ 16‐199).

### Isolation of peripheral blood mononuclear cells

Peripheral blood mononuclear cells (PBMC) from patients and HCs were isolated from fresh, EDTA blood by Ficoll density gradient centrifugation. EDTA blood was diluted with phosphate‐buffered saline (PBS) in a 1:1 ratio, layered over the same volume of Biocoll Separating Solution (Bio&Sell, Nuremberg, Germany), and centrifuged for 25 minutes at 800 × *g* at room temperature. Thereafter, the PBMC fraction was isolated and washed twice using 0.5% bovine serum albumin in PBS.

### Flow cytometry

PBMC were pretreated with Human TruStain FcX (BioLegend, San Diego, USA) to block Fc‐receptors and stained for surface markers by incubation for 15 minutes at 4°C in the dark with the fluorescently labeled antibodies that are listed in Supplementary Table 3. After surface staining, PBMCs were fixed and permeabilized with the True Nuclear Buffer kit (Biolegend) according to the manufacturer's protocol and stained with anti‐FoxP3, anti‐Helios, and anti‐Ki‐67 antibodies (Supplementary Table 3). To quantify surface expression of CD25 by flow cytometry, QuantiBRITE PE beads (BD biosciences, San Jose, USA) were used. Data were acquired using the FACSCanto II (BD) or the CytoFlex S Flow Cytometer (Beckman Coulter, Brea, USA) and analyzed using FlowJo v10 (BD) or Kaluza v2.3 (Beckman Coulter).

### In vitro IL‐2 stimulation of PBMC

Isolated PBMCs from patients with PM/DM (2 × 10^6^/mL) were cultured in RPMI‐1640 (Bio&SELL, Feucht, Germany) and supplemented with 10% fetal calf serum (FCS), 1% penicillin, and 1% streptomycin at 37°C with 5% CO_2_. PBMCs were stimulated for 24 hours with either 1, 5, or 10 ng/mL of recombinant human IL‐2 (aldesleukin; Proleukin, Novartis, Nuremberg, Germany) or left unstimulated. PBMC were then stained and analyzed by flow cytometry (n = 8) or were prepared for messenger RNA (mRNA) isolation (n = 6).

### Quantitative real‐time polymerase chain reaction

Total RNA was isolated from PBMCs by using the NucleoSpin RNA II Kit (Macherey‐Nagel, Dueren, Germany), and complementary DNA (cDNA) was synthesized by using the iScript cDNA Synthesis Kit (Bio‐Rad, Hercules, USA) according to the manufacturer's instructions. Relative expression levels of *IL2RA*, *IL2RB*, *IL2RB*, *ENTPD1*, *IKZF2*, and *CTLA4* genes were determined by using the SsoAdvanced Universal SYBR Green Supermix (Bio‐Rad, Hercules, USA) and the respective primer pairs (see Supplementary Table 4 for primers and Supplementary Table 5 for real‐time polymerase chain reaction setup). The relative quantification method 2^−∆∆Ct^ was applied to normalize the expression levels of samples to *GAPDH* and *18S rRNA* as reference genes and to obtain fold change values.

### Low‐dose IL‐2 treatment of patients with PM/DM

One patient with refractory dermatomyositis (patient 1) and one with refractory polymyositis (patient 2) (see Supplementary Table 6 for baseline characteristics) underwent low‐dose IL‐2 therapy at our site between February 2024 and April 2024 as an add‐on to pre‐existing standard‐of‐care treatment within a compassionate use setting after having provided written informed consent following ICH/GCP guidelines and the Declaration of Helsinki. The low‐dose IL‐2 regimen consisted of daily subcutaneous injections of 1.5 million IU of recombinant human IL‐2 (aldesleukin; Proleukin, Novartis, Germany) for five consecutive days (induction phase: days 0–4), followed by a maintenance phase with seven weekly injections of 1.5 million IU until the end of week seven (day 49). The end‐of‐treatment (EOT) visit was scheduled at day 56 (week 8), and the follow‐up visit was four weeks thereafter at day 84. Changes from baseline in relevant laboratory parameters and in Treg frequencies and counts were assessed at days 5 (1 day after the 5‐day induction phase), 14, 28, 42, 56, and 84. PBMCs were isolated and analyzed by flow cytometry according to the procedures described earlier. Clinical responses were assessed by the ACR/EULAR response criteria in adult PM and DM according to the Total Improvement Score (TIS),^23^ which was calculated from the changes in the six core set measures between the baseline visit and the EOT visit at day 56 (week 8) using the web‐based calculator: https://www.niehs.nih.gov/research/resources/imacs/response_criteria/adult.html.

### Statistical analysis

Statistical analysis was performed with GraphPad Prism software (La Jolla, USA). Nonparametric Mann‐Whitney *U* test was used to assess for differences in immune cells between patients and HCs. Nonparametric correlation analysis was performed by determining Spearman's rank correlation coefficient.

Data were screened for extreme values using the robust regression and outlier removal method (false discovery rate Q = 1 %). Any values flagged as outliers were excluded from the correlation analyses. A paired analysis of variance (ANOVA) test was applied when the assumption of normality was met. When normality was not given, a nonparametric Friedman test was conducted for paired groups. A Shapiro‐Wilk test was applied to test for the normality of data distribution and differences between groups. Data are presented as mean ± SD or as median with interquartile range (IQR). Differences were considered significant if *P* values were <0.05. Data sets used in this study including anonymized individual data are available from the corresponding author upon reasonable request.

## RESULTS

### Treg from patients with PM/DM exhibit an IL‐2 deprived phenotype that is aggravated in active disease

Insufficient availability of IL‐2 causes dysregulation of Treg homeostasis due to a lack of growth and survival signals.^7^, ^8^, ^15^ Because IL‐2 induces expression of the IL‐2 receptor α‐chain CD25, loss of surface expression of CD25 on Treg and the resulting decline in the highly suppressive CD25hi Treg subset are considered phenotypic signs of IL‐2 deficiency as previously described in patients with SLE.^14^, ^21^ Analysis of peripheral blood Treg by flow cytometry showed that the percentages of total FoxP3+CD127lo Treg among CD3+CD4+ T cells were comparable between patients with PM/DM and HC (Figure 1A). By contrast, analysis of surface expression of CD25 on Treg from patients with PM/DM revealed substantially lower frequencies of Treg expressing CD25 at high (CD25hi subset; *P* = 0.0052) and intermediate levels (CD25int subset; *P* = 0.0493) and a reciprocal increase in CD25 negative cells (CD25− subset; *P* = 0.0074) compared with HC (Figure 1B). The reduction in CD25hi Treg was mostly pronounced in patients with active myositis having elevated levels of the CK above the upper limit of normal (*P* = 0.0036 vs HC) (Figure 1C). No significant differences in the frequencies of Helios+, Ki67+, or CD39+ cells among FoxP3+CD127lo Treg were evident between PM/DM and HC (Supplementary Figure 1A–C). Similarly, there were no relevant differences in the frequencies of CD45RO‐CCR7+ naïve, CD45RO+CCR7+ central memory (CM)‐like, CD45RO+CCR7− effector/memory (EM)‐like, and CD45RO‐CCR7− terminally differentiated effector/memory (TEM)‐like Treg between PM/DM and HC (Supplementary Figure 1D). Analysis of CD3+CD4+FoxP3− conventional T cells (Tcon) revealed no differences in the frequencies of proliferating Ki67+ Tcon, the ratio between Ki67+Treg and Ki67+ Tcon, in the frequencies of CXCR5+ and PD‐1+ Tcon, and in the distribution of naïve, CM, EM, or TEM Tcon when all patients with PM/DM were compared with HC (Supplementary Figure 2A–E). However, frequencies of CD45RO+CCR7− EM Tcon were significantly higher in patients with active versus inactive myositis (*P* = 0.0449), resulting in a lower ratio between CD25hi Treg and EM Tcon (*P* = 0.0171 vs HC) (Supplementary Figure 2F and G). Overall, these findings suggest that disease activity is associated with a dysregulation of the Treg–IL‐2 axis and an increased generation of EM Tcon, reflecting an imbalance between CD25hi Treg and effector Tcon, which is considered a typical feature of autoimmune diseases.^13^, ^21^, ^24^, ^25^

**Figure 1:**
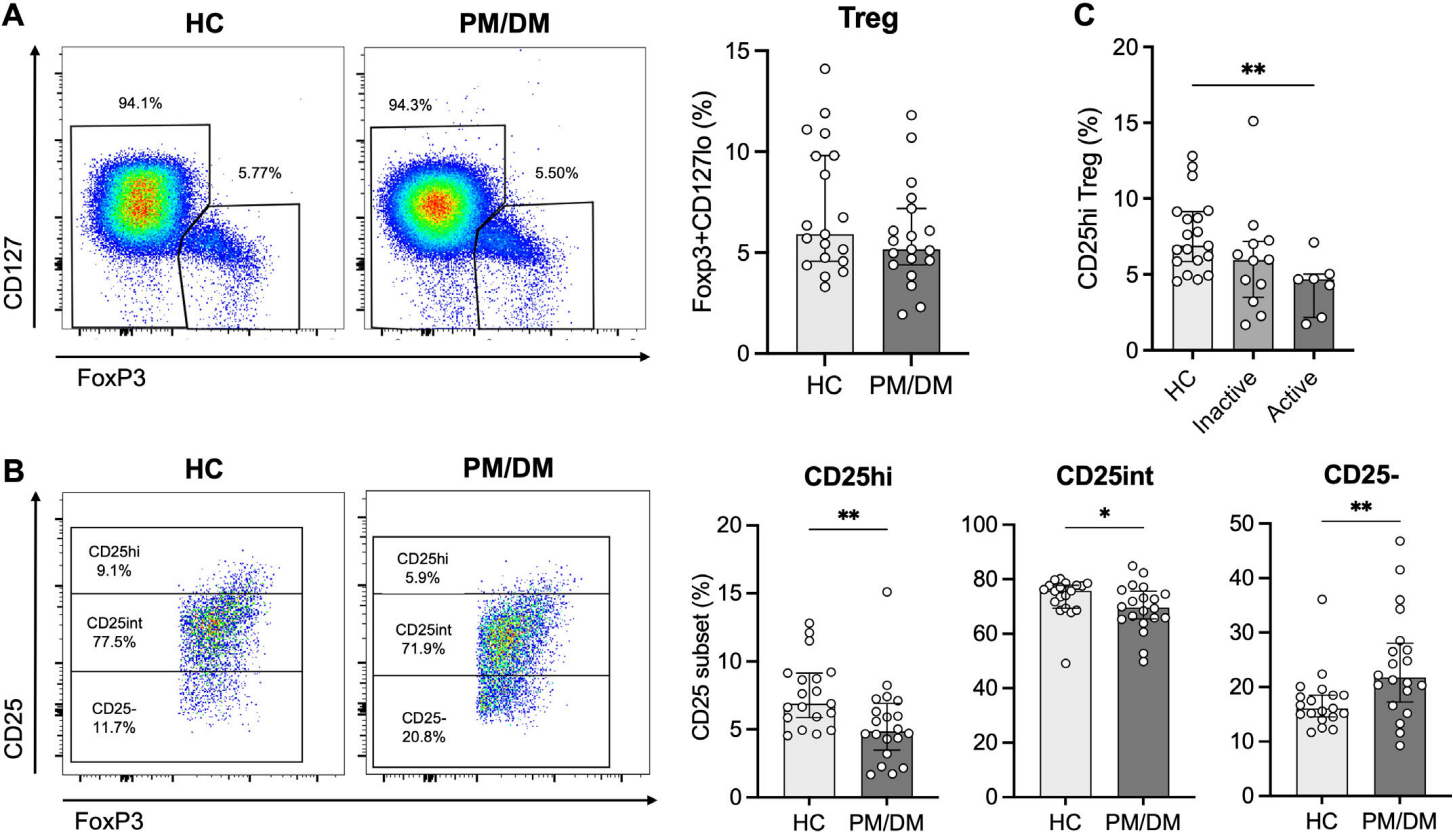
IL‐2 deprived state of Treg in PM/DM. (A) Representative dot plots and bar diagram indicate the frequencies of FoxP3+CD127lo Treg among CD3+CD4+ T cells from HCs (n = 19) and patients with PM/DM (n = 20) analyzed by flow cytometry. (B) Representative dot plots and bar diagrams show the frequencies of CD25hi, CD25int, and CD25− cell subsets among CD3+CD4+FoxP3+CD127lo Treg from HC and patients with PM/DM. Percentages in the dot plots indicate the designated population among the parent population. (C) Frequencies of CD25hi Treg in HC, patients with PM/DM with inactive myositis (n = 12), and patients with PM/DM with active myositis (n = 7). Data are medians with IQR. Mann‐Whitney test was used to compare differences between groups (*P < 0.05, **P < 0.01). DM, dermatomyositis; HC, healthy control; IL‐2, interleukin‐2; IQR, interquartile range; PM, polymyositis.

### Functionally competent and thymic‐derived Treg are enriched in the CD25hi subset in both patients with PM/DM and HCs

Next, we aimed to investigate whether loss of CD25 affects the activity or functional state of the Treg population in patients with PM/DM and whether there are phenotypic differences among the CD25hi, CD25int, and CD25− Treg subsets compared with HCs. Hence, we analyzed expression of the proliferation marker Ki67 of CD39, an ectonucleotidase involved in Treg‐mediated suppression via extracellular ATP hydrolysis,^26^ and of Helios, a transcription factor predominately expressed in thymic‐derived, lineage stable Treg,^27^, ^28^ across the CD25hi, CD25int, and CD25− Treg subsets. Similar to previous observations in SLE,^14^ there were only minor and rather negligible differences in the distribution of these markers among Treg subsets between patients with PM/DM and HC (Supplementary Figure 3). Nevertheless, and in line with previous findings in SLE,^14^ we found that the proportions of proliferating, Ki67+ cells among Treg were by far highest in the CD25hi Treg subset (Figure 2A), and substantially higher proportions of CD25hi Treg expressed CD39 compared with the CD25int and CD25− Treg subsets (Figure 2B). Expression of Helios was also highest in the CD25hi Treg subset and lowest in the CD25− subset with a similar distribution pattern between patients and HC (Figure 2C). The enrichment of Ki67+, CD39+, and Helios+ cells in the CD25hi Treg subset indicates an association between expression levels of CD25 and the activity and functional state of Treg. Loss of the CD25hi Treg subset, as observed in PM/DM, thus leads to a relevant shortage of functionally competent and activated Treg capable of sufficiently counteracting autoimmune responses.

**Figure 2:**
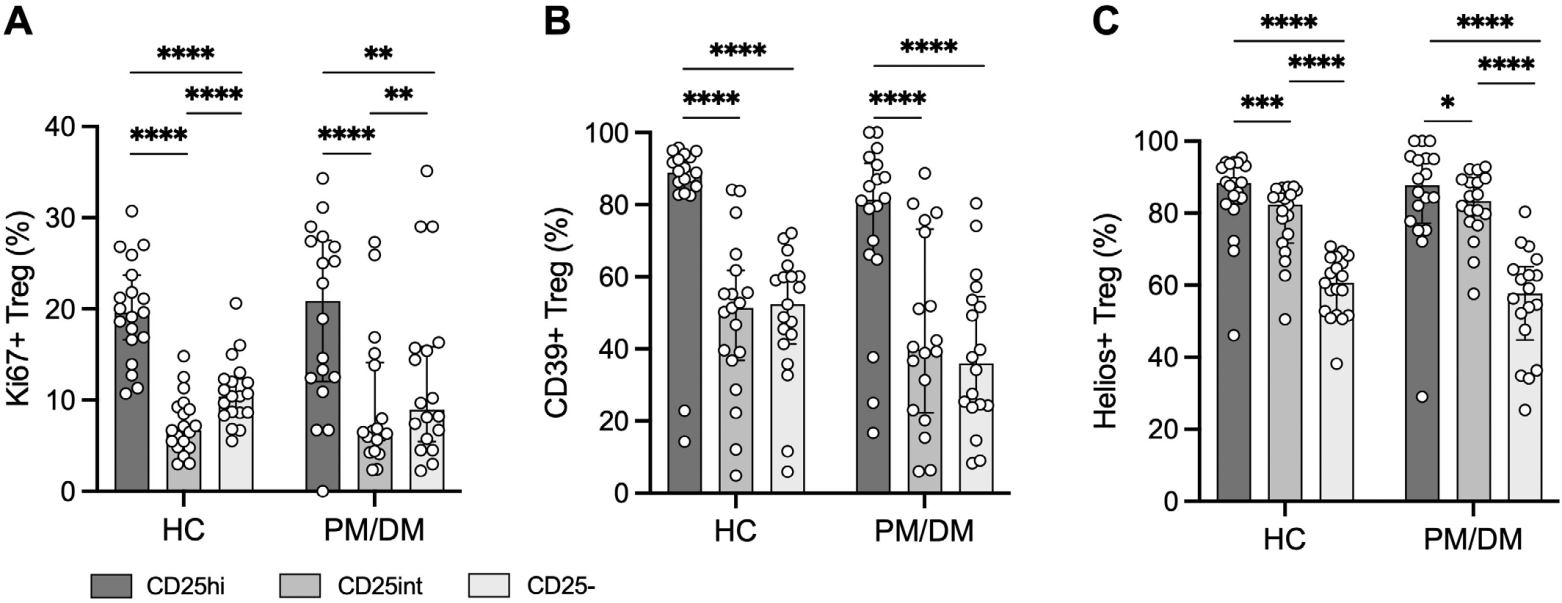
Functionally competent and thymic‐derived Treg are enriched in the CD25hi Treg subset. CD3+CD4+Foxp3+CD127lo Treg from patients with PM/DM and HC were analyzed by flow cytometry and gated according to the CD25 expression levels. Bar diagrams show frequencies of Ki‐67+ cells (A), CD39+ cells (B), and Helios + cells (C), among the CD25hi, CD25int, and CD25− Treg subsets from patients with PM/DM (n = 18–20) and HC (n = 19). Data are medians with IQR. Mann‐Whitney test was used to compare HC and PM/DM samples. Wilcoxon signed‐rank test was used for analyzing differences between Treg subsets within each group. (*P < 0.05, **P < 0.01, ***P < 0.001, ****P < 0.0001). DM, dermatomyositis; HC, healthy control; IQR, interquartile range; PM, polymyositis.

### CXCR5+ follicular Treg are diminished in active PM/DM

Follicular Treg (TfR), defined by CXCR5 and/or PD‐1 expression, are a specialized subset of FoxP3+ Treg that can migrate to lymphoid follicles and suppress germinal center (GC) reactions. A deficiency or dysfunction of TfR could thus facilitate autoimmune responses in GCs.^29^, ^30^, ^31^ We observed a noticeable trend toward reduced frequencies of CXCR5+ TfR cells among Treg in patients with PM/DM compared with HC (*P* = 0.0661) (Figure 3A and B), whereas there was no difference in the frequencies of PD1+ or CXCR5+PD1+ Treg (Figure 3C and 3D). Of note, similar to CD25hi Treg, the reduction in CXCR5+ TfR cells was most pronounced in patients with active myositis (*P* = 0.0335 vs HC) (Figure 3E). Complementary correlation analyses between T cell subsets and clinical biomarkers revealed several associations in patients with PM/DM: an inverse correlation between CD39+ Treg and CXCR5+ Tfh cells and a positive correlation between CD39+ Treg and Ki67+ Treg that were not observed in HC. There were also correlation trends between C‐reactive protein and CD25+ Treg (inverse) or CD25− Treg (positive) and a positive correlation between Ki67+CD25hi Treg and sIL‐2R (detailed description of results and data are presented in the Supplementary Material in conjunction with Supplementary Figure 4).

**Figure 3:**
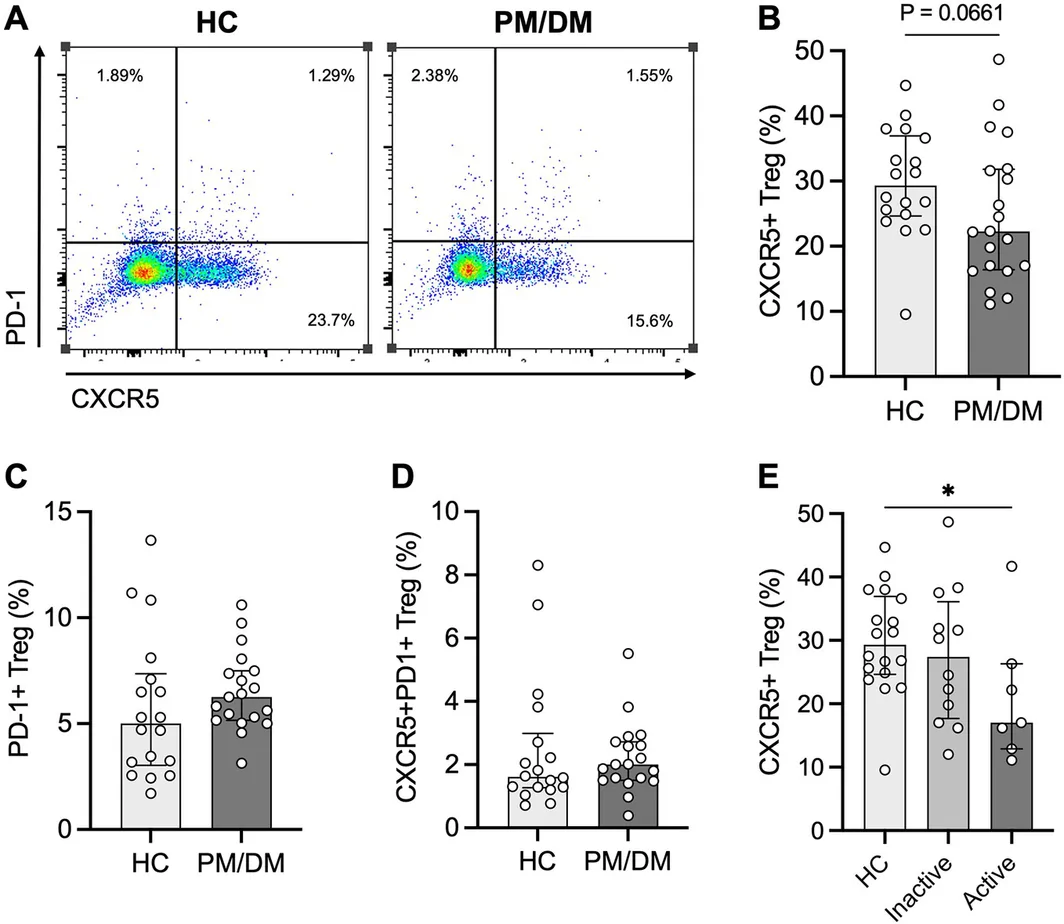
Reduced frequencies of CXCR5+ follicular Treg in active PM/DM. Representative dot plots (A) and bar diagrams show the frequencies of CXCR5+ (B), PD‐1+ (C), and of CXCR5+PD‐1+ (D) cells among CD3+CD4+FoxP3+CD127lo gated Treg from patients with PM/DM (n = 19–20) compared with HC (n = 18). (E) Frequencies of CXCR5+ Treg in HC, patients with PM/DM with inactive myositis (n = 12), and patients with PM/DM with active myositis (n = 7). Data are medians with IQR. Mann‐Whitney test was used to compare differences between groups (*P < 0.05). DM, dermatomyositis; HC, healthy control; IQR, interquartile range; PM, polymyositis. Color figure can be viewed in the online issue, which is available at http://onlinelibrary.wiley.com/doi/10.1002/art.70151/abstract.

### In vitro stimulation with low doses of IL‐2 selectively recovers CD25hi Treg and enhances expression of immunoregulatory molecules

To test whether low doses of IL‐2 are capable of enhancing CD25 expression and recovering the CD25hi Treg subset in PM/DM, PBMC from patients with PM/DM were stimulated in vitro with 1 or 10 ng/mL of recombinant human IL‐2 (aldesleukin) for 24 hours, and frequencies of CD25‐expressing Treg subsets as well as the quantity of CD25 molecules were determined by flow cytometry. Stimulation with 1 ng/mL of IL‐2 increased the frequencies of the CD25hi Treg subset (Figure 4A) and the quantity of CD25 molecules on CD25+ and CD25hi Treg (Figure 4B), with no additional effect observed at the higher concentration (10 ng/mL). No increase in the amount of CD25 molecules was observed on CD4+FoxP3− Tcon at either concentration (Figure 4B), indicating that IL‐2 selectively augments CD25 expression in Treg, even at higher concentrations.

**Figure 4:**
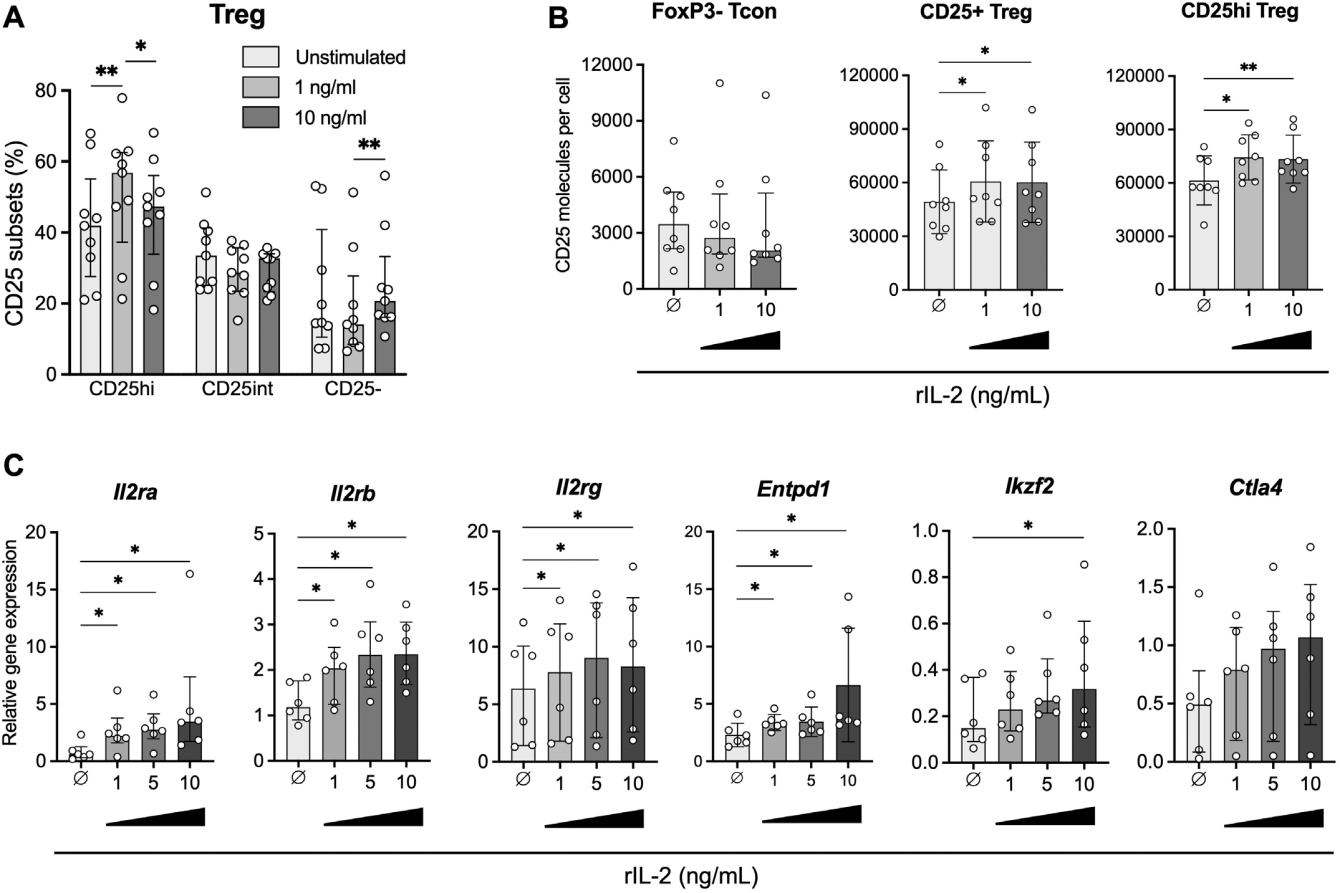
Recovery of CD25hi Treg and induction of immunoregulatory molecules by in vitro stimulation with low doses of IL‐2 in patients with PM/DM. Frequencies of CD25hi, CD25int, and CD25− Treg subsets among CD3+CD4+FoxP3+CD127lo Treg (A) and the quantity of CD25 molecules per cell in CD3+CD4+FoxP3− Tcon, CD3+CD4+FoxP3+CD127loCD25+ Treg, and CD3+CD4+FoxP3+CD127loCD25hi Treg (B) were determined by flow cytometry in patients with PM/DM (n = 8) following in vitro stimulation for 24 hours with none, 1 ng/mL, and 10 ng/mL of rIL‐2. (C) Relative mRNA expression levels of the three IL‐2 receptor chains CD25 (*IL2RA*), CD122 (*IL2RB*) and CD132 (*IL2RG*), and of the immune regulatory molecules CD39 (*ENTPD1*), Helios (*IKZF2*) and CD152 (*CTLA4*) were quantified by qPCR (normalized to GAPDH/18S rRNA) in patients with PM/DM (n = 6) following in vitro stimulation for 24 hours with none, 1 ng/mL, 5 ng/mL, and 10 ng/mL of rIL‐2. Data are mean ± SD. A paired ANOVA test was applied for normally distributed data sets, and a nonparametric paired Friedman test was used for non‐normally distributed data sets (*P < 0.05, **P < 0.01). ANOVA, analysis of variance; DM, dermatomyositis; HC, healthy control; IQR, interquartile range; mRNA, messenger RNA; PM, polymyositis; qPCR, quantitative polymerase chain reaction; rIL‐2, recombinant human IL‐2.

To obtain complementary insights into the effects of in vitro low‐dose IL‐2 stimulation at a transcriptional level, we quantified the mRNA expression of several immunoregulatory molecules in PBMC from patients with PM/DM after stimulation with 1, 5, and 10 ng/mL of IL‐2 for 24 hours. Stimulation with all concentrations markedly increased mRNA expression of the three IL‐2 receptor chains CD25 (*IL2RA*), CD122 (*IL2RB*), and CD132 (*IL2RG*), and of CD39 (*ENTPD1*) compared with unstimulated controls. Moreover, IL‐2 augmented Helios (*IKZF2*) expression, mostly pronounced at the highest dose, and to a lesser extent also upregulated CD152 (*CTLA4*) expression (Figure 4C). In contrast to protein‐level analysis of CD25, mRNA expression of all three IL‐2 receptor chains, as well as Helios and CD152, increased moderately in a dose‐dependent manner (Figure 4C), suggesting a differential regulation of transcription and protein expression. Overall, these data propose that in vitro stimulation with low doses of IL‐2 selectively recovers Treg activity and induces a favorable immunoregulatory expression pattern in PBMC from patients with PM/DM, providing a further rationale for the clinical application of low‐dose IL‐2 therapy in patients with PM/DM.

### Low‐dose IL‐2 therapy expands CD25hi Treg and improves clinical outcomes in two patients with refractory PM/DM

To provide a first in vivo proof‐of‐concept for the reversibility of Treg defects and the clinical efficacy of low‐dose IL‐2 therapy in PM/DM, we treated two patients with refractory PM/DM and active myositis using an established regimen of low‐dose IL‐2 therapy as add‐on therapy to pre‐existing treatment.^19^ At baseline (day 0), these patients exhibited persistent elevations of CK, myoglobin, and aldolase, reflecting chronically active myositis that was accompanied by progressive muscle pain and weakness, along with increased Troponin T levels indicative of active myocardial involvement (Supplementary Table 6). Changes from baseline in laboratory parameters, Treg frequencies and counts, and clinical responses assessed by the TIS^23^ were determined throughout the treatment (days 0, 5, 14, 28, 42, and 56) and the follow‐up (day 84) period.

Low‐dose IL‐2 therapy was very well‐tolerated, with adverse events limited to mild (grade 1) injection site reactions, fatigue, and insomnia, consistent with reports from previous clinical trials investigating low‐dose IL‐2 therapy in different autoimmune diseases.^11^, ^12^, ^18^, ^19^, ^25^, ^32^, ^33^ Both patients showed a 3.5‐ and 3.7‐fold increase, respectively, in the proportions of CD25hi Treg among CD3+CD4+FoxP3+CD127lo Treg, along with increased frequencies of Ki67+ and CD39+ Treg after the induction phase on day 5 (Figure 5A). In patient 2, this was accompanied by an increase in both the frequency and counts of CD3+CD4+CD127loCD25+ Treg, whereas in patient 1, the increase in CD3+CD4+CD127loCD25+ Treg was delayed and became evident only by day 14 of the treatment phase. During the maintenance phase, Treg frequencies and counts remained stable above baseline, with both patients showing an approximately 2‐fold increase from baseline by the EOT phase at day 56 (week 8) (Figure 5B and C). A transient increase in CD16+CD56+ NK cell counts and an initial drop in CD19+ B cell counts were observed after the induction phase, whereas total CD3+CD4+ and CD3+CD8+ T cells showed only minor increases that went along with a general increase in lymphocyte counts (Supplementary Figure 5), indicating that low‐dose IL‐2 therapy predominantly expanded the Treg population.

**Figure 5:**
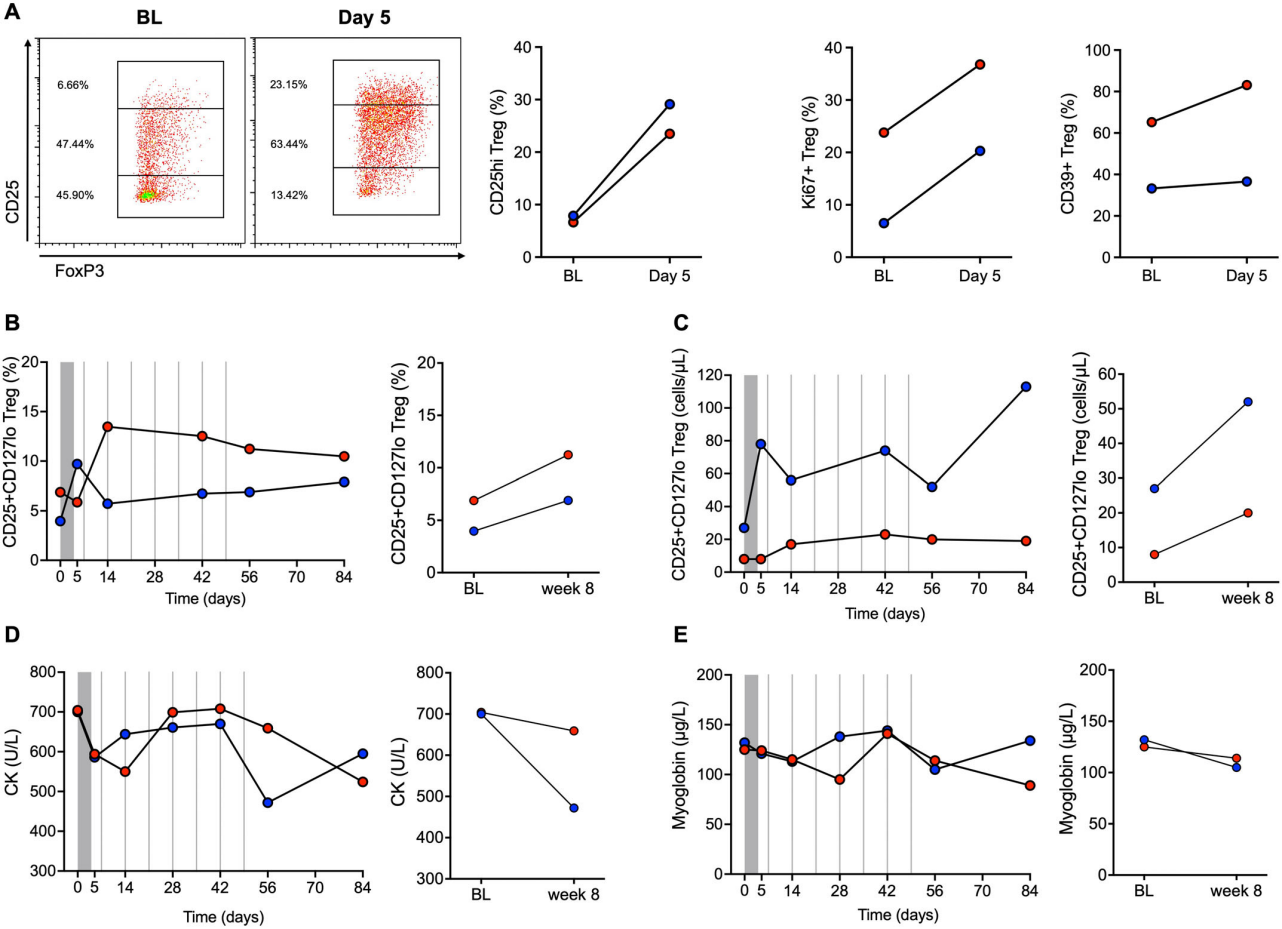
Changes in Treg and clinical biomarkers during low‐dose IL‐2 treatment of two patients with PM/DM. Two patients with refractory PM/DM were treated with a low‐dose IL‐2 regimen for eight weeks and followed‐up for another four weeks. (A) Representative dot plots (left) show changes in Treg subsets according to CD25 expression levels in CD3+CD4+FoxP3+CD127lo gated Treg from one patient with PM/DM at BL and one day after the five‐day induction cycle (day 5). Right panel shows changes in the frequencies of CD25hi, Ki67+, and CD39+ Treg among CD3+CD4+FoxP3+CD127lo gated Treg from baseline to day five in the two treated patients. Longitudinal changes during the eight‐week treatment phase (until day 56, week 8) and the post‐treatment follow‐up phase (until day 84, left panels) and changes from baseline to week 8 (right panels) in the frequencies (B) and absolute counts (C) of CD25+CD127lo Treg among CD3+CD4+ T cells. Longitudinal changes during treatment and follow‐up phase (left panels) and changes from baseline to week eight (right panels) in levels of CK (D) and myoglobin (E). Red and blue lines denote individual patient trajectories. Laboratory biomarkers and blood samples for flow cytometry were analyzed at the indicated time points. Vertical gray lines represent the time points of IL‐2 administration. BL, baseline; CK, creatine kinase; DM, dermatomyositis; IL‐2, interleukin‐2; PM, polymyositis.

Patient 1 showed a moderate clinical improvement at day 56 with a TIS score of 42.5, and patient 2 had no clinical improvement with a TIS score of 2.5, mainly because of pre‐existing chronic and persistent severe myasthenia and related disabilities (see web‐based TIS calculations in the Supplementary Material). In both patients, however, levels of CK markedly dropped by 15% to 16% during the induction phase, slightly increased again during the maintenance phase, and dropped again at the end of the treatment phase at day 56 (week 8), resulting in a reduction from baseline of 6% in patient 1 and of 33% in patient 2 (Figure 5D). In both patients, levels of myoglobin and aldolase, and levels of Troponin T in patient 1, decreased during the treatment phase (Figure 5E and Figure 6). At the post‐treatment follow‐up visit, levels of CK further declined in patient 1, or, despite a moderate rise, remained below baseline levels in patient 2.

## DISCUSSION

Current knowledge about the nature and origins of an impairment in Treg biology in IM is still limited. Previous investigations characterizing Treg in patients with DM reported reduced numbers of FoxP3+ Treg in skin lesions compared with other skin diseases in parallel to reduced numbers of CD4+FoxP3+CD25+ Treg in peripheral blood compared with HCs.^34^ However, a deeper understanding of the causes of Treg dysregulation in IM has the potential to provide a mechanistically driven rationale for the implementation of low‐dose IL‐2 therapy that aims to recover and expand Treg, as has been explored in SLE.^11^, ^12^, ^14^, ^18^, ^19^, ^32^

One of our key findings is that Treg from patients with PM/DM exhibit a loss of the highly suppressive CD25hi Treg subset along with an increased prevalence of effector Tcon, resulting in an imbalance between Treg and effector Tcon. These phenotypic alterations, which are similar to previous findings in SLE,^14^ were mostly pronounced in patients with active disease and resemble typical hallmarks for an insufficient availability of the essential Treg growth and survival factor IL‐2. Phenotypic comparison of the CD25hi, CD25int, and CD25− Treg subsets from patients with PM/DM and HCs confirmed that activated and thymic‐derived Treg with a presumably high suppressive capacity are enriched within the CD25hi Treg subset and that such enrichment is principally independent of the health state, similar to what was shown in previous SLE studies.^14^ Given this constellation, we conclude that the loss of the CD25hi Treg subset in patients with PM/DM, which is likely to be caused by an acquired shortage of IL‐2, leads to a reduced availability of the one Treg subset that is considered to be most efficient in counteracting autoimmune pathology.

A further noticeable phenotypic alteration was the reduced prevalence of CXCR5+ TfR cells, which was also most pronounced in patients having active myositis. Such a deficiency of CXCR5+ TfR cells could potentially reduce the capacity of Treg cells for homing to lymphoid follicles to suppress GC reactions and B cell differentiation, representing an additional mechanism of immune dysregulation in autoimmune diseases, which should be explored in more depth in future research.

These results prompted us to investigate whether the IL‐2 deprived and exhausted state of Treg seen in PM/DM can be reversed by stimulation with low doses of IL‐2. We found that in vitro stimulation with the lowest concentration of IL‐2 was already capable of recovering CD25hi Treg and inducing a favorable immunoregulatory expression pattern. The reversibility of these Treg defects thus provided an important rationale for the clinical translation of low‐dose IL‐2 therapy in PM/DM. On this basis, we could indeed provide preliminary evidence for the favorable clinical and biologic effects of low‐dose IL‐2 therapy in PM/DM, as shown by the decrease in muscle enzymes and the clinical improvement that was accompanied by a robust increase in CD25hi Treg after treatment of two refractory patients with a low‐dose IL‐2 regimen. Our findings are also in line with a recent work that reported decreases in CK and lactate dehydrogenase in patients with PM/DM who were undergoing a five‐day treatment with low doses of IL‐2.^35^

One limitation of our study is its exploratory nature because of the relatively low number of patients, particularly the small number of only seven patients with active myositis, which restricted the identification of relevant correlations between biomarkers of disease activity and immunologic parameters. Another limitation is the pilot character of our treatment approach, which included only two refractory patients and thus does not provide a robust level of confidence beyond an early proof‐of‐concept stage. Future research should also aim to address potential differences in the suppressive function of Treg and mechanistically prove that deficiency for IL‐2 is responsible for the observed Treg impairment in IM. Nevertheless, our findings stimulated the implementation of an investigator‐initiated, randomized, and placebo‐controlled basket trial that aimed to investigate the clinical efficacy and mode‐of‐action of low‐dose IL‐2 therapy in four different chronic inflammatory diseases, including patients with PM/DM (EU Clinical Trial Number: 2023‐510297‐14).

In summary, our data suggest that Treg exhaustion, which is most likely due to shortage of IL‐2, is pathophysiologically relevant in PM/DM. Reconstitution of the “fitness” of Treg by low‐dose IL‐2 therapy could thus be a promising targeted treatment strategy in PM/DM.

## AUTHOR CONTRIBUTIONS

All authors contributed to at least one of the following manuscript preparation roles: conceptualization AND/OR methodology, software, investigation, formal analysis, data curation, visualization, and validation AND drafting or reviewing/editing the final draft. As corresponding author, Dr Humrich confirms that all authors have provided the final approval of the version to be published and takes responsibility for the affirmations regarding article submission (eg, not under consideration by another journal), the integrity of the data presented, and the statements regarding compliance with institutional review board/Declaration of Helsinki requirements.

## Supporting information


**Disclosure form**.

**Supplementary Figure S1:** Phenotype and distribution of Treg subsets in PM/DM
**Supplementary Figure S2:** Phenotype and distribution of Tcon subsets in PM/DM
**Supplementary Figure S3:** Comparisons of Treg subsets according to CD25 expression between patients with PM/DM and HC
**Supplementary Figure S4:** Correlations between T cell subsets and clinical biomarkers
**Supplementary Figure S5:** Changes in lymphocyte subsets during low‐dose IL‐2 treatment of two patients with PM/DM
**Supplementary Figure S6:** Changes in clinical biomarkers during low‐dose IL‐2 treatment of two patients with PM/DM.
**Supplementary Table S1:** Summary of clinical and laboratory characteristics of patients with PM/DM undergoing immunophenotyping
**Supplementary Table S2:** Individual demographics, clinical characteristics and disease‐related therapies of patients with PM/DM undergoing immunophenotyping
**Supplementary Table S3:** Antibody conjugates and clones used for flow cytometry
**Supplementary Table S4:** Primers used for real‐time PCR
**Supplementary Table S5:** Real‐time PCR setup
**Supplementary Table S6:** Demographics, clinical characteristics and concomitant and previous therapies of the two patients undergoing low‐dose IL‐2 therapy
