# Supplementary material for: Interleukin‐2 Deprived State of Regulatory T cells and Their Recovery by Low‐Dose Interleukin‐2 in Patients With Inflammatory Myopathies
Source: Arthritis Rheumatol. 2026 Apr 28;78(11):2341–50. doi: 10.1002/art.70151 (PMC13712568; doi:10.1002/art.70151)
Supplement: Supplementary file 1 [file ART-78-2341-s001.pdf]

Supplementary Material

Interleukin-2 deprived state of regulatory T cells and their recovery by low-dose interleukin-2 in patients with inflammatory myopathies

Justus Ohmes, PhD[1#], Luisa R. Monne, MD[1#], Sara Comdühr, PhD[1], Fynn Gerlach, M.Sc[1], Hanna Grasshoff, MD[1], Alexander Dübbers, MD[1], Antje Müller, PhD[1], Alexander Scheffold, PhD[2], Gabriela Riemekasten, MD[1], Reza Akbarzadeh, PhD[1*], Jens Y. Humrich, MD[1*]

Table of contents

1. Supplementary **Figure S1**. Phenotype and distribution of Treg subsets in PM/DM
2. Supplementary **Figure S2**. Phenotype and distribution of Tcon subsets in PM/DM
3. Supplementary **Figure S3**. Comparisons of Treg subsets according to CD25 expression between patients with PM/DM and HC
4. Supplementary **Figure S4**. Correlations between T cell subsets and clinical biomarkers
5. Supplementary **Figure S5**. Changes in lymphocyte subsets during low-dose IL-2 treatment of two patients with PM/DM
6. Supplementary **Figure S6**. Changes in clinical biomarkers during low-dose IL-2 treatment of two patients with PM/DM.
7. Supplementary **Table S1**. Summary of clinical and laboratory characteristics of patients with PM/DM undergoing immunophenotyping
8. Supplementary **Table S2**. Individual demographics, clinical characteristics and disease-related therapies of patients with PM/DM undergoing immunophenotyping
9. Supplementary **Table S3**. Antibody conjugates and clones used for flow cytometry
10. Supplementary **Table S4.** Primers used for real-time PCR
11. Supplementary **Table S5**. Real-time PCR setup
12. Supplementary **Table S6.** Demographics, clinical characteristics and concomitant and previous therapies of the two patients undergoing low-dose IL-2 therapy
13. **Gating strategy** for flow cytometry
14. **Web-based calculations of Total Improvement Scores (TIS)** for the two patients undergoing low-dose IL-2 therapy

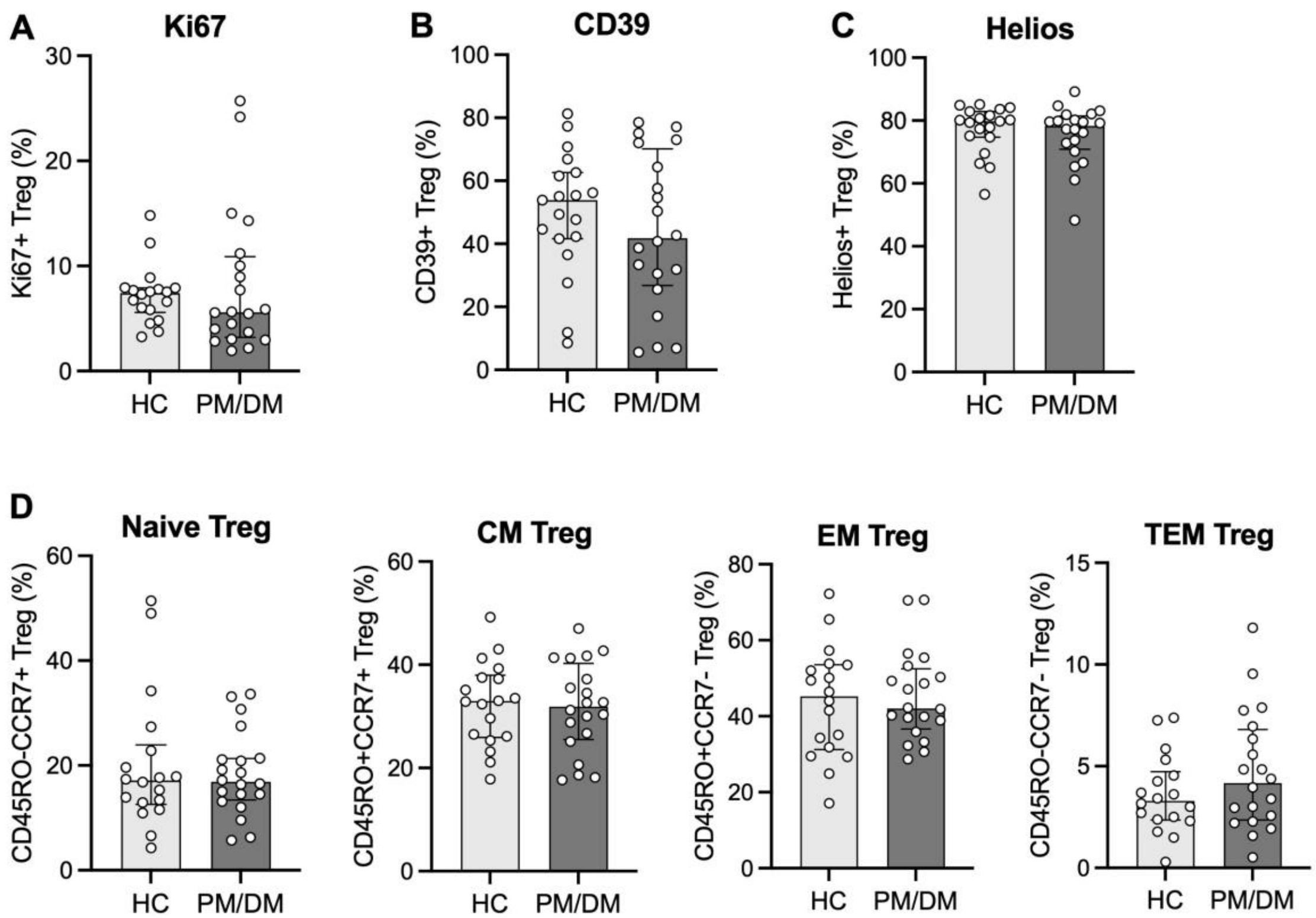

Supplementary Figure S1. Phenotype and distribution of Treg subsets in PM/DM. Frequencies of Ki67+ (**A**), CD39+ (**B**), and Helios+ (**C**) cells among CD3+CD4+FoxP3+CD127lo Treg, and the distribution of CD45RO-CCR7+ naive, CD45RO+CCR7+ central memory-like (CM), CD45RO+CCR7- effector memory-like (EM) and CD45RO-CCR7- terminally differentiated effector memory-like (TEM) Treg among CD3+CD4+FoxP3+CD127lo Treg (**D**) from healthy controls (HC; n=19) and patients with PM/DM (n=18-20) were determined by flow cytometry. Data are medians with IQR. Mann-Whitney test was used for statistical analyses.

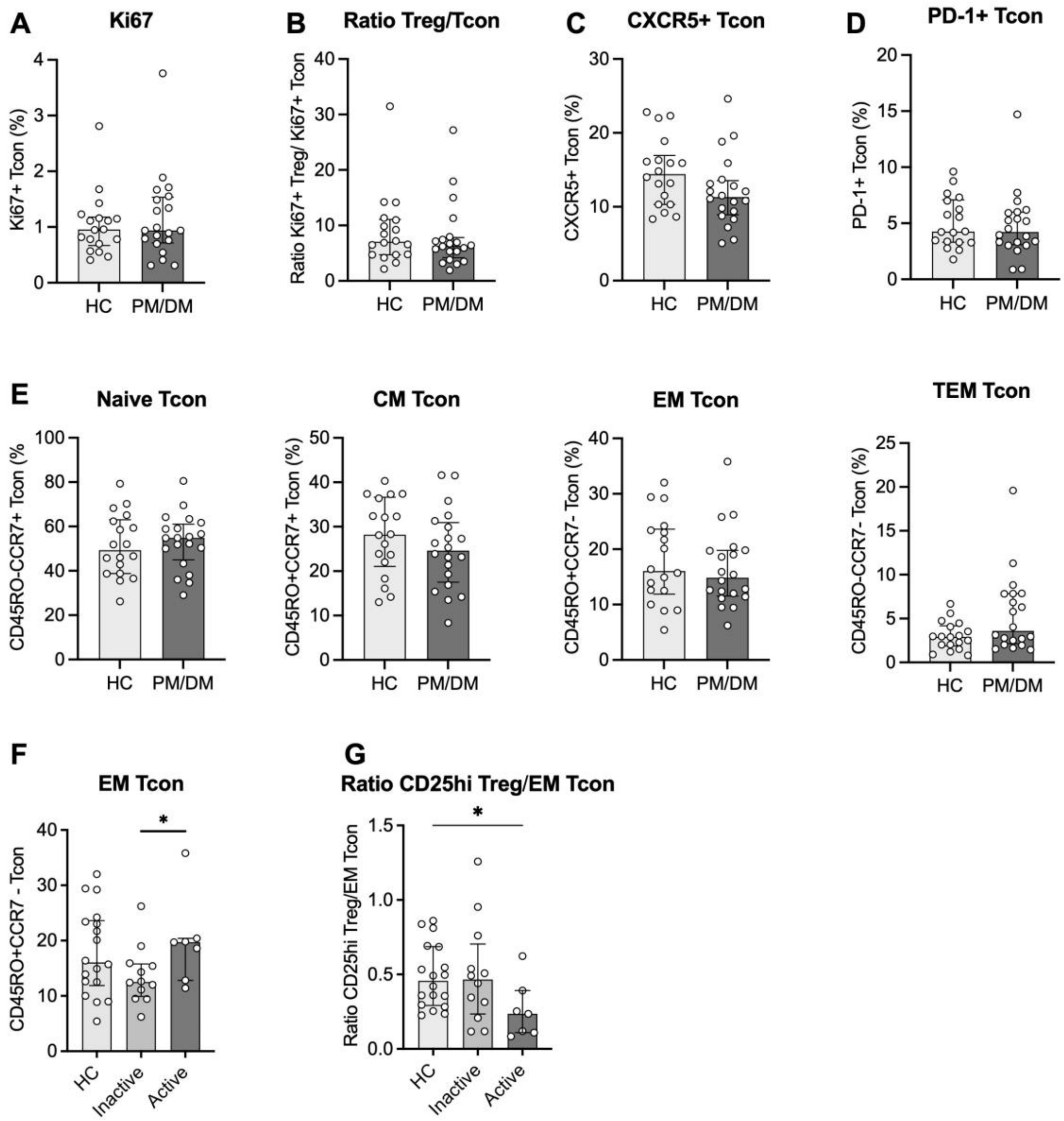

Supplementary Figure S2. Phenotype and distribution of Tcon subsets in PM/DM. **A-E**) Frequencies of Ki67+ cells among CD3+CD4+FoxP3- Tcon (**A**), the calculated ratio between frequencies of Ki67+ Treg and Ki67+ Tcon (**B**), frequencies of CXCR5+ (**C**) and of PD-1+ (**D**) cells among CD3+CD4+FoxP3- Tcon, and the distribution of CD45RO-CCR7+ naive, CD45RO+CCR7+ central memory (CM), CD45RO+CCR7- effector memory (EM) and CD45RO-CCR7- terminally differentiated effector memory (TEM) Tcon among CD3+CD4+FoxP3- Tcon (**E**) in healthy controls (HC; n=19) and patients with PM/DM (n=18-20) were determined by flow cytometry. **F, G**) Frequencies of CD45RO+CCR7- EM Tcon (**F**), and the calculated ratio between frequencies of CD25hi Treg and EM Tcon (**G**) in HC (n=19), PM/DM patients with inactive myositis (n=12), and PM/DM patients with active myositis (n=7). Data are medians with IQR. Mann-Whitney test was used for statistical analyses (*p<0.05).

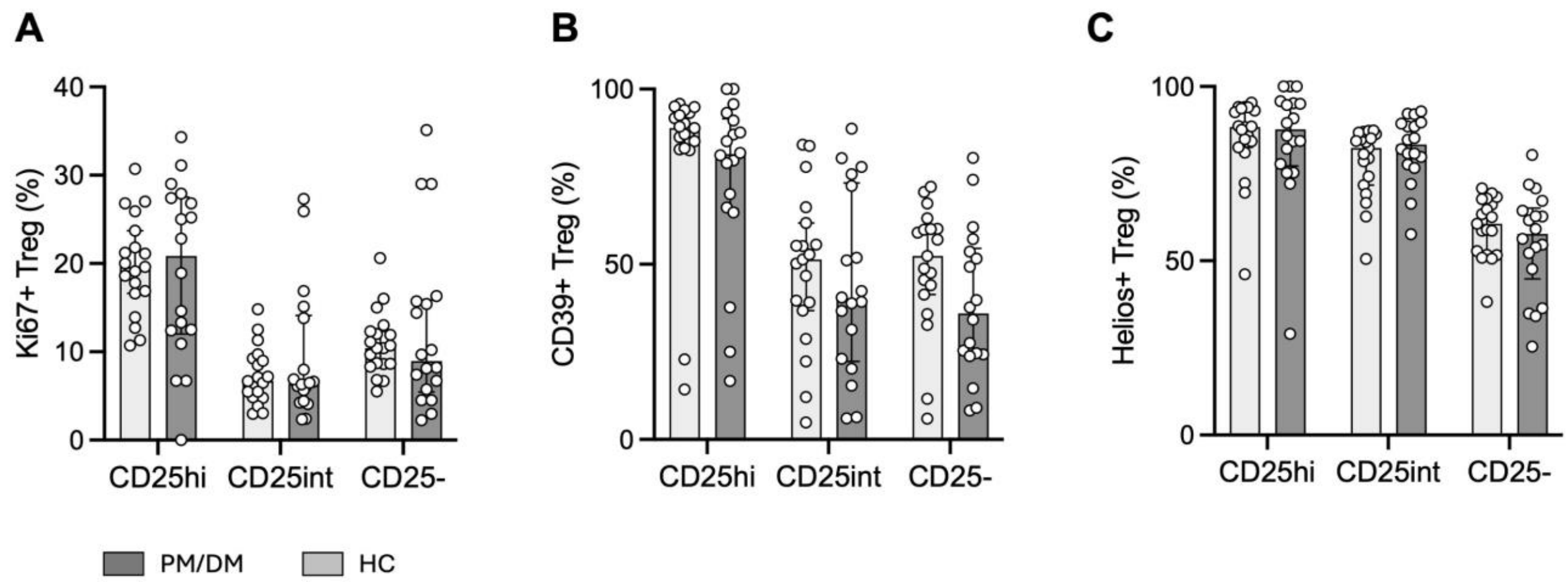

Supplementary Figure S3. Comparisons of Treg subsets according to CD25 expression between patients with PM/DM and HC. Frequencies of Ki67+ cells (**A**), CD39+ cells (**B**), and Helios+ cells (**C**) among the CD25hi, CD25int, and CD25- Treg subsets from healthy controls (HC; n=19) and patients with PM/DM (n=18-20) were determined by flow cytometry. Data are medians with IQR. Mann-Whitney test was used for statistical analyses.

Interrelationship between T cell subsets and clinical biomarkers of disease activity in PM/DM

Correlation analyses between different T cell subsets from PM/DM patients revealed a significant inverse correlation between frequencies of CD39+ Treg and CXCR5+ follicular helper Tcon (Tfh) (r=-0.45, p=0.0446; Fig. S4A), suggesting that lower availability of functionally active Treg may facilitate enhanced differentiation of pro-inflammatory Tfh cells. In line with the assumption of the relationship between Treg activation and function, there was a positive correlation between frequencies of Ki67+ Treg and CD39+Treg (r=0.58; p=0.0075; Fig. S4B). Interestingly, no such correlations were observed in HC (Fig S4A, B), and no other relevant correlations between T cell subsets were evident.

Next, we performed correlation analyses between T cell subsets and laboratory biomarkers of disease activity. Although the majority of analyzed patients had C-reactive protein (CRP) values within normal ranges, we found a trend towards a moderate inverse correlation between frequencies of CD25+ Treg and CRP levels (r=-0.41; p=0.0793; Fig. S4C), and vice versa, a trend towards a moderate positive correlation between frequencies of CD25- Treg and CRP levels (r=0.40; p=0.0858; Fig. S4D), suggesting that a higher extend of the CD25 low, putatively IL-2 deprived, Treg state is associated with a higher level of inflammation. Although, levels of soluble CD25 (soluble IL-2 receptor or sIL-2R) only modestly correlated with the frequencies of total FoxP3+CD127lo Treg (r=0.58; p=0.108; Fig. S4E), there was a strong positive correlation between sIL-2R levels and the frequencies of highly activated and proliferating Ki67+CD25hi Treg (r=0.73; p=0.0311; Fig. S4F), proposing that sIL-2R could be a biomarker of Treg activity in patients with PM/DM. Apart from a positive correlation between levels of IgM and CD25hi Treg (r=0.6903; p=0.0156; data not shown), there were no relevant correlations between T cell subsets and levels of CK, Glutamic-Oxaloacetic Transaminase (GOT), Glutamic pyruvic transaminase (GPT), Lactate Dehydrogenase (LDH), Ferritin, IgA or IgG, which could be a result of the rather low number of patients having active myositis (n=7) within our cohort.

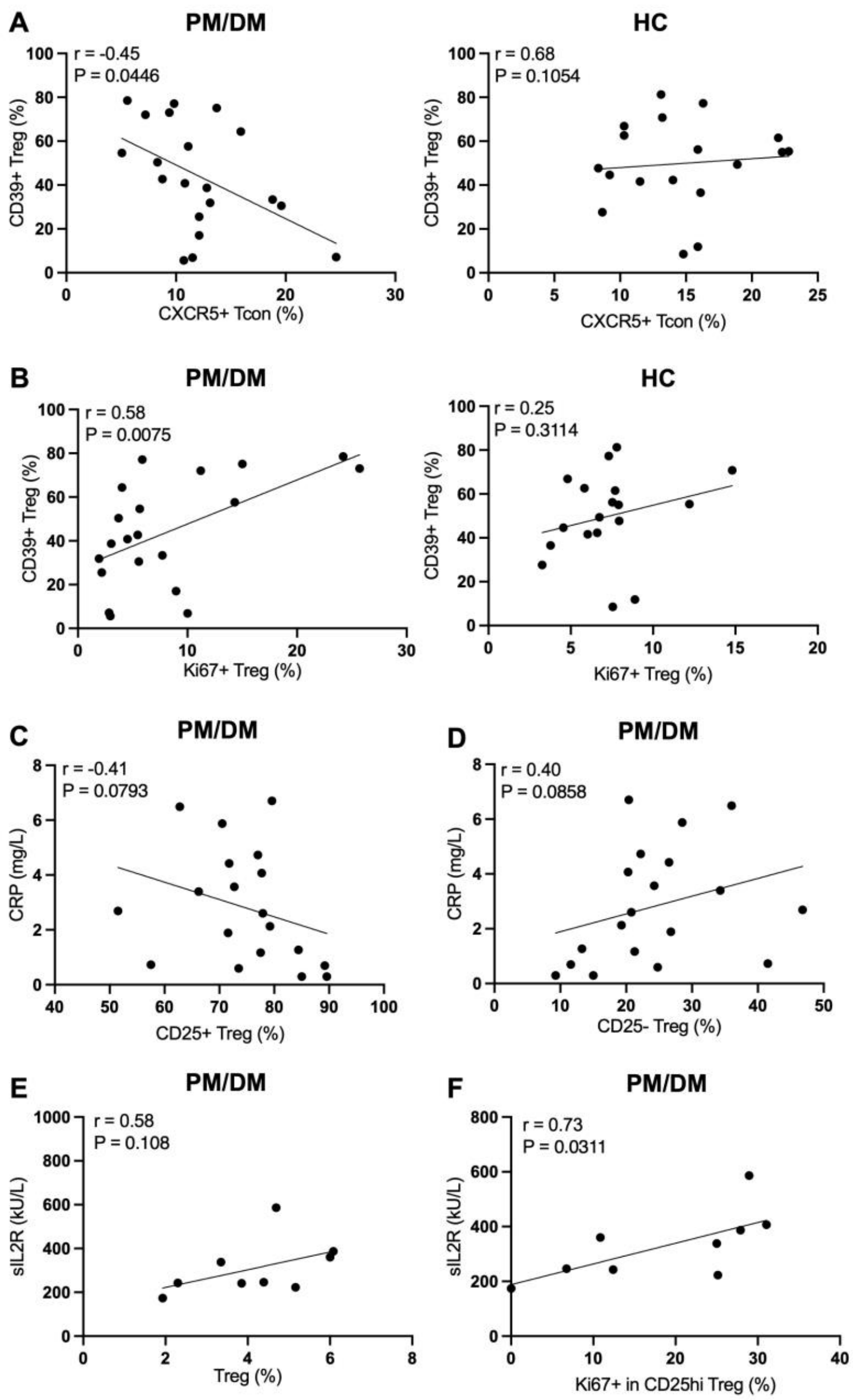

Supplementary Figure S4. Correlations between T cell subsets and clinical biomarkers.

Graphs show the correlations between frequencies of CD39+ cells among CD3+CD4+FoxP3+CD127lo Treg and CXCR5+ cells among CD3+CD4+ FoxP3- Tcon in PM/DM and HC (**A**), between frequencies of CD39+ cells among CD3+CD4+FoxP3+CD127lo Treg and Ki67+ cells among CD3+CD4+FoxP3+CD127lo Treg in PM/DM and HC (**B**), between plasma levels of C-reactive protein (CRP) and frequencies of CD25+ cells among CD3+CD4+FoxP3+CD127lo Treg (**C**), between plasma levels of CRP and frequencies of CD25- cells among CD3+CD4+FoxP3+CD127lo Treg (**D**), between plasma levels of soluble IL-2 receptor (sIL-2R) and frequencies of FoxP3+CD127lo Treg among CD3+CD4+ T cells (**E**), and between plasma levels of sIL-2R and frequencies of Ki67+ cells among CD3+CD4+FoxP3+CD127loCD25hi Treg (**F**). Spearman's rank correlation coefficients were used. Correlation coefficients (r), p values (P) and linear regression lines are indicated in the respective graphs. One outlier was excluded from the correlation analyses with sIL-2R levels due to an extreme value of sIL-2R (ROUT method).

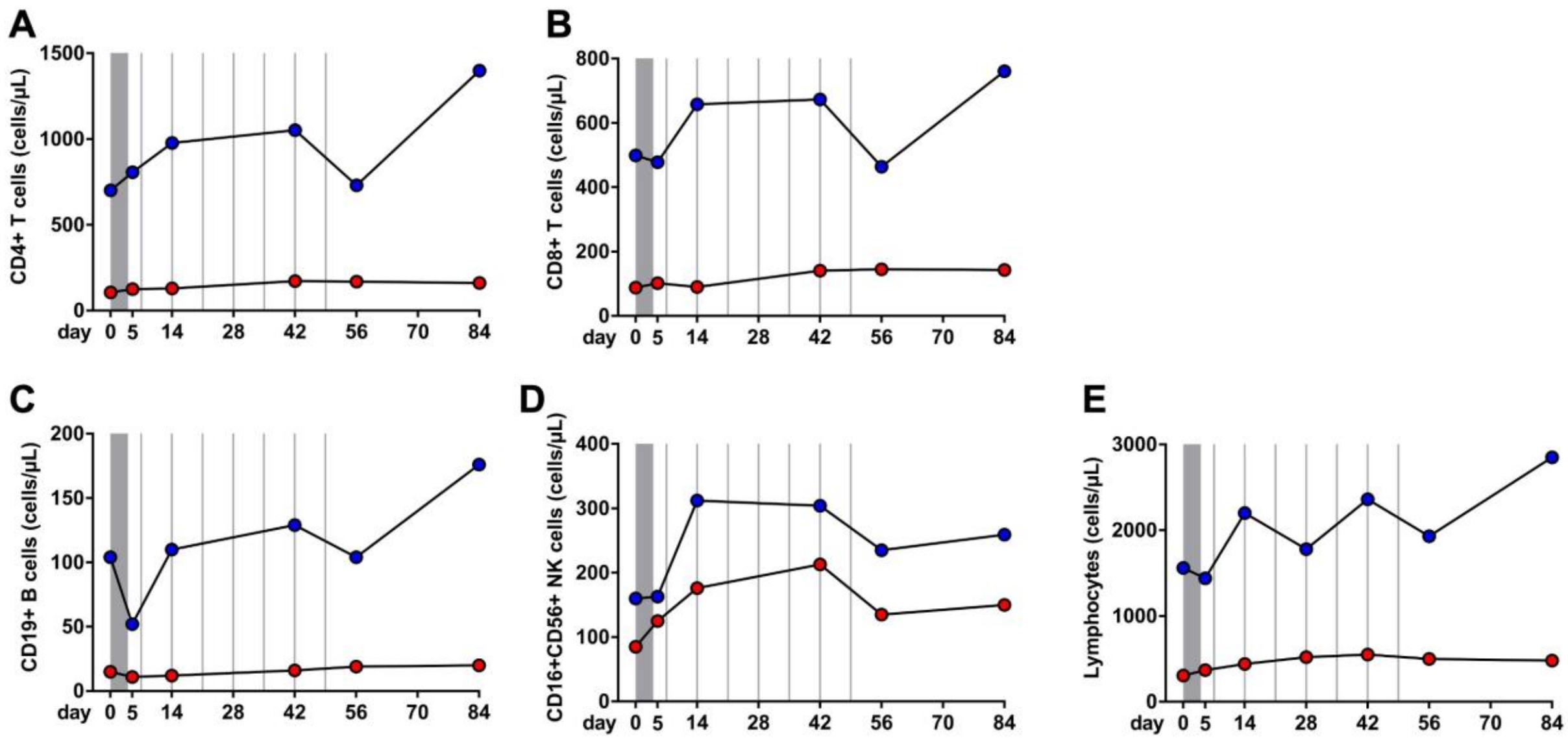

Supplementary Figure S5. Changes in lymphocyte subsets during low-dose IL-2 treatment of two patients with PM/DM. Two patients with refractory PM/DM were treated with a low-dose IL-2 regimen consisting of a 5-day induction cycle (day 0 to day 4) with daily s.c. injections of 1.5 Mio IU of IL-2 (aldesleukin) followed by 7 weekly s.c. injections of 1.5 Mio IU of IL-2 from day 7 to day 49. Longitudinal changes during the 8-week treatment phase (until day 56, week 8) and the post-treatment follow-up phase (until day 84) in the absolute counts of CD3+CD4+ T cells (**A**), CD3+CD8+ T cells (**B**), CD19+ B cells (**C**), CD16+CD56+ NK cells (**D**) and total lymphocytes (**E**). Red and blue lines denote individual patient trajectories. Blood samples were analyzed by flow cytometry at the indicated time points. Vertical gray lines represent the time points of IL-2 administration.

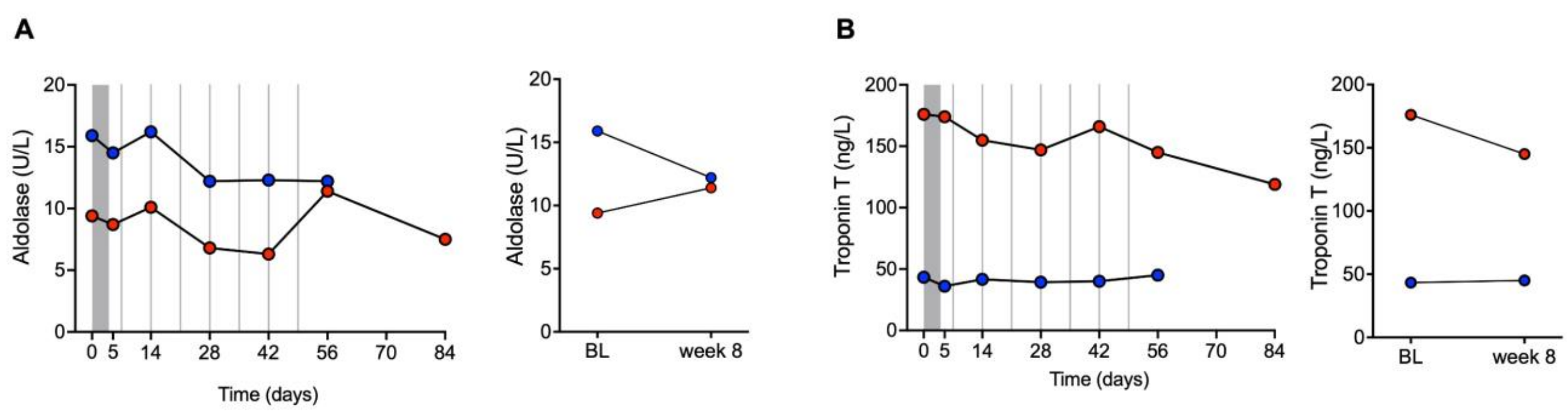

Supplementary Figure S6. **Changes in clinical biomarkers during low-dose IL-2 treatment of two patients with PM/DM**. Two patients with refractory PM/DM were treated with a low-dose IL-2 regimen for 8 weeks and followed-up for another 4 weeks. Longitudinal changes during the 8-week treatment and the post-treatment follow-up phase (left panels) and changes from baseline to week 8 (right panels) in levels of aldolase (**A**), and troponin T (**B**). Red and blue lines denote individual patient trajectories. Laboratory biomarkers and blood samples for flow cytometry were analyzed at the indicated time points. Vertical gray lines represent the time points of IL-2 administration.

Clinical characteristics	Total (n=20)
Female, n (%)	14 (70)
Age [years], median (range)	56 (21-81)
Polymyositis, n (%)	12 (60)
Dermatomyositis, n (%)	8 (40)
Active myositis, n (%)	7 (35)
**Laboratory characteristics**	
CK [U/L], median (range)	154 (41 - 1001)
CRP [mg/L], median (range)	2.65 (<0.3 - 31)
sIL-2R [kU/L], median (range)	349 (174 - 2624)
Lymphocytes [/nL], median (range)	0.89 (0.44 – 2.83)
LDH [U/L], median (range)	222 (177 - 479)
GPT [U/L], median (range)	31 (9 - 94)
GOT [U/L], median (range)	33 (19 - 79)

Supplementary Table S1. Summary of clinical and laboratory characteristics of patients with PM/DM undergoing immunophenotyping. CK - creatine kinase, CRP - C-reactive protein, sIL-2R - soluble IL-2 receptor, LDH - Lactate Dehydrogenase, GPT - Glutamic Pyruvic Transaminase, GOT - Glutamic-Oxaloacetic Transaminase.

P. Nr	Sex [F/M]	Age [y]	Entity	Disease duration [years]	Autoantibody profile	Active myositis [Y/N]	Plasma levels of CK [U/L]	Other clinical features and relevant comorbidities	PM/DM-related therapy	Dose of GC [mg/d]
1	M	70	PM	11	PM/Scl-75<br>Jo-1	N	178	Muscle atrophy, myasthenia, PNP<br>Overlap RA	MTX	1
2	F	64	PM	19	Ku	N	137	Muscle atrophy, myalgia, myasthenia,	HCQ, MMF	2.5
3	M	55	PM	1	Ro52<br>PM/Scl-75	**Y**	**378**	Muscle atrophy, RS, thrombocytopenia	MTX, CSA	0
4	F	81	PM	2	Ro52	**Y**	**605**	Muscle atrophy, myocarditis, amyloidosis	RTX, CSA	4
5	M	63	DM	3	Ro52<br>Mi-2	N	118	Myalgia, myasthenia, Gottron´s papules	None	7.5
6	F	21	DM	1	PM-Scl-75/100<br>Ro52	N	92	Peri-/myocarditis, pleuritis, arthritis, facial erythema, Gottron´s papules	RTX, MMF<br>CSA	3
7	M	31	PM	1	ANA	N/A	N/A	Myalgia, arthralgia	CSA	0
8	F	58	DM	0	PM/Scl-100<br>Scl-70	N	168	Myalgia, facial erythema, mechanic hands	HCQ	0
9	F	66	DM	1	ANA, TIF-1g	N	73	Muscle atrophy, myasthenia / tetraplegia, myalgia, PNP, dysphagia, myocarditis, dermatitis<br>Overlap RA	RTX, MMF<br>CSA, HCQ	15
10	F	77	PM	2	Ro52	N	125	Myalgia, calcinosis cutis, dysphagia, RS, Sicca-Syndrome<br>Overlap RA	RTX, HCQ	5
11	F	64	DM<br>ASyS	4	ANA, Ro52,<br>PL-7	N	165	ILD, mechanic hands, facial erythema, alopecia, oral ulcers, RS	MMF, CSA	0
12	M	42	PM	0	Ku, PL-7<br>PL-12	**Y**	**528**	Myalgia, fasciculations, arthritis	None	0
13	M	32	PM	5	ANA, Ro52<br>PM/Scl-100	**Y**	**601**	Myalgia, myasthenia, myocarditis, ILD, polyserositis, calcinosis cutis<br>Overlap SSc	RTX, CSA	20
14	F	50	PM<br>ASyS	13	ANA, Jo-1	N	117	Myalgia, ILD, arthritis, RS	RTX, MMF	2
15	F	76	DM<br>ASyS	4	ANA, Jo-1,<br>MDA-5	N	108	Myalgia, myasthenia, ILD, dermatitis, periorbital edema, Gottron´s papules, PNP, arthritis, RS, Sicca-Syndrome<br>Overlap RA	RTX, MMF	1
16	F	60	DM	2	ANA, AMA-M2	**Y**	**247**	Myalgia, myasthenia, dermatitis, oral ulcers, arthritis<br>Overlap RA, PBC	MTX, HCQ	12.5
17	F	53	PM	1	PM/Scl-75	N	143	Myalgia, myasthenia, arthritis<br>Overlap RA	MTX, CSA	4
18	F	49	DM	1	ANA, Mi-2b,<br>SRP	**Y**	**1001**	Myalgia, peri-/myocarditis, facial erythema, Gottron´s papules, RS	RTX, MMF	20
19	F	56	PM	0	ANA, TIF-1g	**Y**	**922**	Myalgia, myasthenia, muscle atrophy	None	0
20	F	50	PM	0	ANA, Mi-2b<br>Ku, NXP-2	N	41	Myalgia, myasthenia, arthritis,	None	7.5

Supplementary Table S2. Individual demographics, clinical characteristics and disease-related therapies of patients with PM/DM undergoing immunophenotyping.

ASyS - Antisynthetase-Syndrome, CSA - ciclosporin A, GC - glucocorticoids, HCQ - hydroxychloroquine, ILD - interstitial lung disease, MMF - mycophenolate mofetil, MTX - methotrexate, N/A - not assessable, PBC - primary biliary cholangitis, PNP - polyneuropathy, RA - rheumatoid arthritis, RS - Raynaud´s syndrome, RTX - rituximab, SSc - systemic sclerosis, y - years.

Antigen	Conjugate	Clone	Supplier
CD3	APC/Cy7	UCHT1	Biolegend, San Diego, USA
CD4	PerCp	RPA-T4	Biolegend, San Diego, USA
CD127	BV510	A019D5	Biolegend, San Diego, USA
CD39	PE-Cy7	A1	Biolegend, San Diego, USA
CD45RO	BV510	UCHL1	Biolegend, San Diego, USA
CCR7	PE-Cy7	G043H7	Biolegend, San Diego, USA
CXCR5	PE	J252D4	Biolegend, San Diego, USA
PD-1	AF488	EH12.2H7	Biolegend, San Diego, USA
CD25	PE	2A3	BD Biosciences, San Jose, USA
FoxP3	AF647	206D	Biolegend, San Diego, USA
Helios	AF488	22F6	Biolegend, San Diego, USA
Ki-67	BV421	Ki-67	Biolegend, San Diego, USA

Supplementary Table S3. Antibody conjugates and clones used for flow cytometry.

Gene	Primer sequences	Amplicon size
IL2RA	F: GAGATCCCACACGCCACATT<br>R: TGTTCCGAGTGGCAGAGCTT	181 bp
IL2RB	F: CATGTCTCAGCCAGGGCTTC<br>R: TGGACTTGGCAGGAAGTGTC	232 bp
IL2RG	F: GGATGGGCAGAAACGCTACA<br>R: ATTCGGGGCATCGTCCGTT	231 bp
ENTPD1	F: ACTATCGAGTCCCCAGATAATGC<br>R: CCTGATCCTTCCCATAGCACAA	97 bp
HELIOS	F: CCTCCGTTTATCTTCAGCCCG<br>R: GTGAAAGCTCATTGTCACACGTTAT	151 bp
CTLA4	F: AGGCAACGGAACCCAGATTT<br>R: GCTCTGTTGGGGGCATTTTC	197 bp
GAPDH	F: GAGTCAACGGATTTGGTCGT<br>R: GGAAGATGGTGATGGGATTT	159 bp
18SRNA	F: GTAACCCGTTGAACCCCATT<br>R: CCATCCAATCGGTAGTAGCG	151 bp

Supplementary Table S4. Primers used for real-time PCR.

Components	Volume [µ]
SYBR Green Mix I	10
Nuclease-free H_2O	7
Primer forward	1
Primer reverse	1
cDNA	1
**Σ**	**20**

Reaction	Time [s]	Temperature [°C]	Cycles
Preliminary denaturation	300	95	1
Denaturation	10	95	
Annealing	15	primer-specific	40
Elongation	20	72	
Melting curve	540	60-95	

Supplementary Table S5. Real-time PCR setup.

P. Nr	Sex	Age [years]	Entity	Disease duration [years]	Auto-antibody profile	Organ involvements	Concomitant therapies	Previous therapies
1	F	42	DM	1	Mi-2	Active myositis, myasthenia, periorbital erythema, Gottron's papules, cardiac involvement (Troponin T elevation)	HCQ, MMF, CSA, GC	CP, IVIG
2	F	52	PM	1	Jo-1 Ro60	Active myositis, myasthenia, cardiac involvement (Troponin T elevation)	HCQ, MTX, GC	AZA, CSA, IVIG

Supplementary Table S6. Demographics, clinical characteristics, and concomitant and previous therapies of the two patients undergoing low-dose IL-2 therapy. Abbreviations: AZA - azathioprine, CSA – ciclosporin A, CP - cyclophosphamide, GC – glucocorticoids, HCQ - hydroxychloroquine, IVIG – intravenous immunoglobulins, MMF - mycophenolate mofetil, MTX - methotrexate.

Gating strategy for flow cytometry

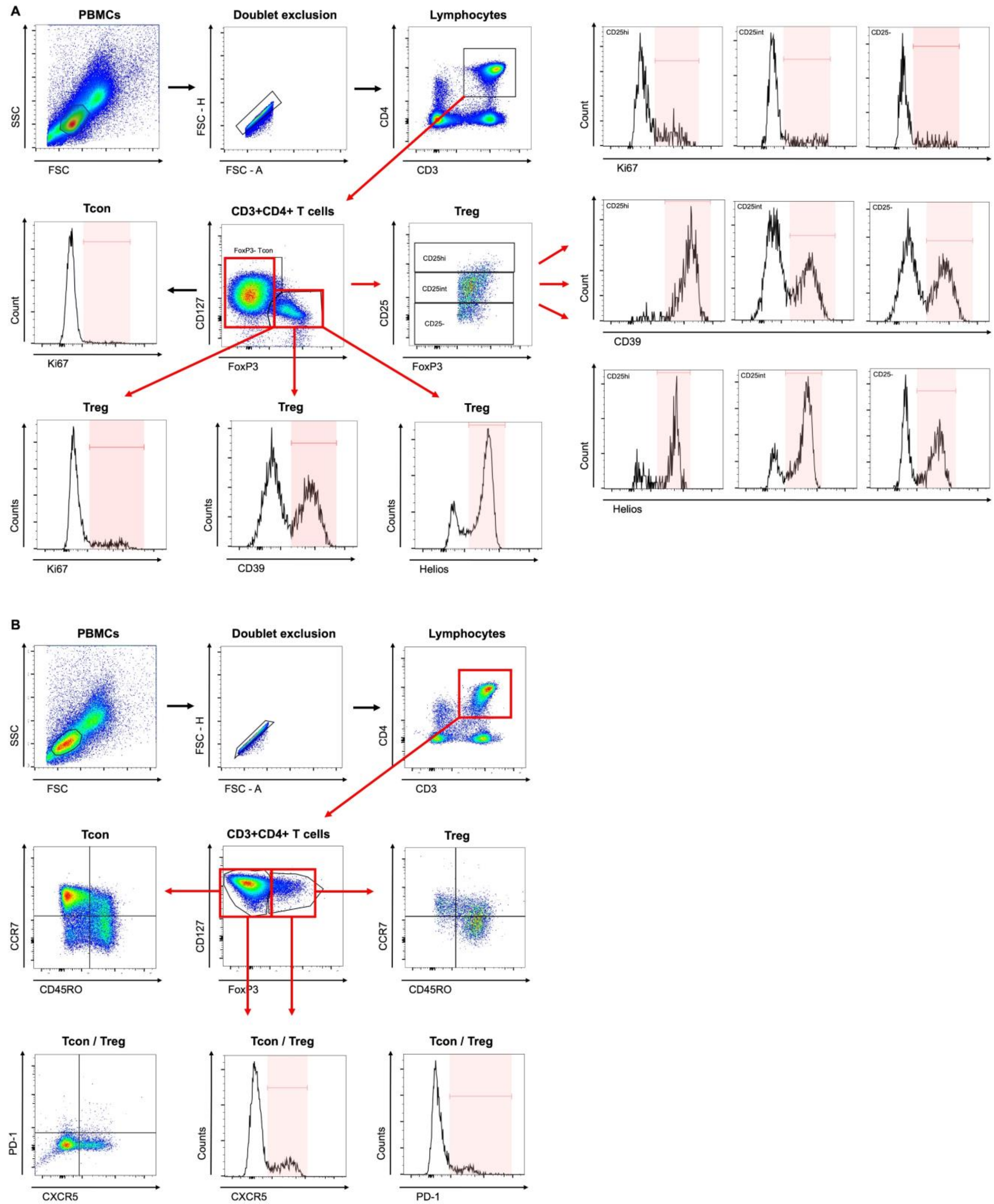

Web calculator for ACR/EULAR Criteria for Minimal, Moderate, and Major Clinical Response in Adult Dermatomyositis and Polymyositis

The Myositis Response Criteria was validated using all 6 core set measures.

Patient 1

At a minimum, you must enter:
1. Physician Global Disease Activity AND,
3. Manual Muscle Testing AND
at least 2 other core set measures in order for a Total Improvement Score to be calculated.

Core Set Measure			BASELINE	FOLLOW-UP	Change	Relative % Change	Absolute % Change	Improvement Score
1. Physician Global Disease Activity [0.0-10.0 cm]			8.0	5.0	3.0	37	30	17.5
2. Patient Global Disease Activity [0.0-10.0 cm]			7.0	3.0	4.0	57	40	7.5
3. Manual Muscle Testing (MMT)	Maximum MMT Score Possible:	80	78	79	1	1	1	0
4. Health Assessment Questionnaire (HAQ) [0.000-3.000]			0.375	0.000	0.38	97	13	5
5. Extra-muscular Disease Activity [0.0-10.0 cm]			3.0	0.5	2.5	83	25	12.5

6. Muscle Enzymes
Please select the UNITS first, and then enter each serum muscle enzyme level tested and the associated upper limit of normal.

◉ **[Unit/L]**
○ **[microkat/L]**

	Upper Limit of Normal	BASELINE	FOLLOW-UP	Change	Relative % Change	Absolute % Change	Improvement Score
Creatine Kinase (CK)	170	704.00	659.00				
Lactate Dehydrogenase (LDH)							
Aspartate Aminotransferase (AST)							
Alanine Aminotransferase (ALT)							
Aldolase							
Calculate Most Abnormal Muscle Enzyme: Creatine Kinase (CK)	170	704	659	45	6	2	0

No Improvement Threshold	< 20
Minimal Improvement Threshold	20
Moderate Improvement Threshold	40
Major Improvement Threshold	60

Total Improvement Score:

42.5 [Moderate Improvement]

Clear Form **Calculate Total Improvement Score**

Web calculator for ACR/EULAR Criteria for Minimal, Moderate, and Major Clinical Response in Adult Dermatomyositis and Polymyositis

The Myositis Response Criteria was validated using all 6 core set measures.

Patient 2

At a minimum, you must enter:
1. Physician Global Disease Activity AND,
3. Manual Muscle Testing AND
at least 2 other core set measures in order for a Total Improvement Score to be calculated.

Core Set Measure		BASELINE	FOLLOW-UP	Change	Relative % Change	Absolute % Change	Improvement Score
1. Physician Global Disease Activity [0.0-10.0 cm]		9.0	8.5	0.5	6	5	0
2. Patient Global Disease Activity [0.0-10.0 cm]		7.0	7.0	0.0	0	0	0
3. Manual Muscle Testing (MMT)	Maximum MMT Score Possible: 80	36	34	-2	-6	-2	0
4. Health Assessment Questionnaire (HAQ) [0.000-3.000]		2.125	2.000	0.13	6	4	0
5. Extra-muscular Disease Activity [0.0-10.0 cm]		3.0	3.0	0.0	0	0	0

6. Muscle Enzymes
Please select the UNITS first, and then enter each serum muscle enzyme level tested and the associated upper limit of normal.

◉ [Unit/L]
○ [microkat/L]

	Upper Limit of Normal	BASELINE	FOLLOW-UP
Creatine Kinase (CK)	170	700.00	472.00
Lactate Dehydrogenase (LDH)			
Aspartate Aminotransferase (AST)			
Alanine Aminotransferase (ALT)			
Aldolase			

Calculate Most Abnormal Muscle Enzyme	Upper Limit of Normal	BASELINE	FOLLOW-UP	Change	Relative % Change	Absolute % Change	Improvement Score
Creatine Kinase (CK)	170	700	472	228	33	9	2.5

Threshold	
No Improvement Threshold	< 20
Minimal Improvement Threshold	20
Moderate Improvement Threshold	40
Major Improvement Threshold	60

Total Improvement Score:

2.5 [No Improvement]

Clear Form

Calculate Total Improvement Score
